# Retraction statement: Dual targeting of RANKL and PD‐1 with a bispecific antibody improves anti‐tumor immunity

**DOI:** 10.1002/cti2.1378

**Published:** 2022-02-23

Dougall WC, Roman Aguilera A, Smyth MJ (2019), Dual targeting of RANKL and PD‐1 with a bispecific antibody improves anti‐tumor immunity. *Clin Transl Immunol*, 8: e01081. https://doi.org/10.1002/cti2.1081. The above article published online on 27 September 2019 in Wiley Online Library (wileyonlinelibrary.com) has been retracted by agreement between the journal's Editor‐in‐Chief, Rajiv Khanna, the Australian and New Zealand Society for Immunology Inc., and John Wiley and Sons Australia, Ltd. The retraction has been agreed following an investigation by the QIMR Berghofer Medical Research Institute into research published by the senior author, Professor Mark J Smyth. The investigation concluded that at least two figures (3b and 4b) generated by the senior author Professor Mark Smyth are likely fabricated. As a result, the conclusions are considered unreliable, and the article has been retracted.

